# Digital toolbox for vector field characterization

**DOI:** 10.1515/nanoph-2021-0484

**Published:** 2021-12-07

**Authors:** Keshaan Singh, Angela Dudley

**Affiliations:** School of Physics, University of the Witwatersrand, Private Bag 3, Johannesburg 2050, South Africa

**Keywords:** digital micro-mirror device, phase retrieval, structured light, wavefront sensing

## Abstract

Vectorial structured light fields have displayed properties advantageous in many disciplines ranging from communications, microscopy and metrology to laser cutting and characterizing quantum channels. The generation of these fields has been made convenient through the implementation of nanophotonic metasurfaces amongst other static and digital techniques. Consequently, the detection and characterisation of these fields is of equal importance. Most existing techniques involve using separate polarization optics and correlation filters to perform the projective measurements – or are only able to perform such measurements on a subset of possible vector states. We present a compact, fully automated measurement technique based on a digital micro-mirror device (DMD), which facilitates the complete, local and global, characterisation of the spatial mode and polarization degrees-of-freedom (DOFs) for arbitrary vectorial fields. We demonstrate our approach through the identification of relevant hybrid-order Poincaré spheres, the reconstruction of state vectors on these spheres, as well as the recovery of the non-separability and states-of-polarization for a variety of vector beams.

## 1 Introduction

The field of structured light focuses on the tailoring of light’s degrees-of-freedom (DOFs) to probe fundamental features of nature as well as to extend the industrial optics toolbox [1], [2], [3]. Prior to the last two decades, disproportionate focus has been given to structuring the intensity and phase DOFs. Recent interest in the tailoring of light’s polarization has led to observations of interesting topological [4, 5] and energy transport [6, 7] behavior – which, in some cases, prove useful for communication [8], metrology [9], microscopy [10] and even laser machining [11]. The increased interest in these structured polarization fields (referred to as vector fields [12]), has unsurprisingly coincided with the development of tools allowing for their convenient generation. These tools take the form of dynamic and geometric phase elements such as nano-structured metasurfaces [13, 14] and liquid crystal q-plates [15]; as well as versatile digital devices such as liquid crystal spatial light modulators (SLMs) [16] and digital micro-mirror devices (DMDs) [17]. Techniques used to characterize these vector fields include Stokes polarimetry measurements capable of recovering the total spatially varying polarization structure. Stokes measurements also have the ability to recover the concurrence based non-separability of the spatial and polarization DOFs, or vector quality factor (VQF) [18]. Of additional importance to characterizing quantum channels using vector fields, is a basis dependent non-separability measurement (VQF′) acquired through polarization projections in conjunction with spatial mode projections [19, 20]. Naturally, the efficient characterization of vector fields needs to grow in conjunction with their increasing popularity. The basis dependent characterizations have been carried out using arrangements including polarization gratings, mode sorters and q-plates, which – while effective – only permit the measurements in a limited subset of spatial mode bases [21, 22]. Recently a more versatile and efficient approach to the basis dependent measurement was proposed, where the polarisation independence of a DMD was exploited – however, this technique requires a set of parallel polarization optics, making the arrangement complex [23]. Only recently have a few publications been dedicated to full Poincaré polarimetry [24, 25] and the adaptive control of vectorial fields via polarization and phase correction [26, 27]. Universal polarisation mapping has been demonstrated with the use of a GRIN lens [24, 25] to map the polarization analyzer states to vectorial fields with very high-precision and single acquisition measurements. However, this requires custom made optics (in the form of a specially designed GRIN lens), restricting the versatility of the measurement device.

We have previously demonstrated digital Stokes measurements, which provides general information about polarization (in)homogeneity, but no modal information [28]. In this work we present a system, based on a DMD capable of performing sequential and simultaneous spatial and polarization measurements on arbitrary vector fields, permitting their full characterization in a rapid, compact and completely automated manner. Our scheme exploits the small separation angle afforded by a 1° Wollaston prism (WP), the polarization independence of DMDs and polarization interference phenomena to achieve its functionality. The ability to simultaneously project into the spatial and polarization DOFs allows us to extract the global relative phase and amplitude between the constituent orthogonally polarized components, while exclusive spatial mode and polarization projections lead to reconstructions of the scalar fields and resulting polarization structure. Previously demonstrated methods which have been used to acquire polarization and phase information have been reliant on interference with reference plane waves leading to cumbersome optical systems [29, 30], while other techniques use light with polarization known *a priori* to probe anisotropic systems [31]. Our system provides a versatile, fast and compact solution to full field characterization without any prior information or reference. We demonstrate the effectiveness of our approach on a variety of vector beams including beams with large orbital angular momentum (OAM), fractional OAM and beams with non-zero radial orders. Our scheme serves as a versatile automated detection solution to complement the growing utilisation of vector fields in research and industrial settings.

## 2 Concept

To represent a vector field, we will adopt the convenient quantum mechanical notation [3]

(1) $$|\Psi 〉=\cos\left(\frac{\alpha }{2}\right){\text{e}}^{\text{i}\beta /2}|\psi_{1}〉|R〉+sin\left(\frac{\alpha }{2}\right){\text{e}}^{-\text{i}\beta /2}|\psi_{2}〉|L〉.$$

**Figure 1:**
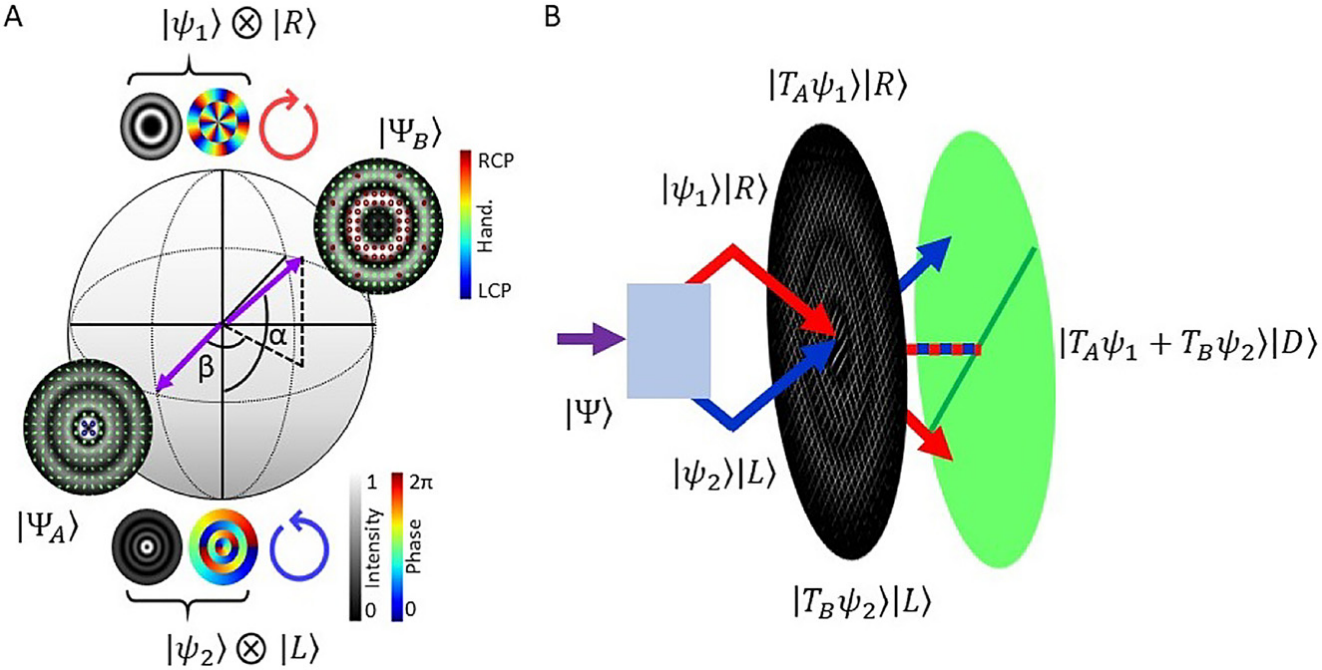
Concept diagrams illustrating our characterization scheme. (A) HOPS with $|\psi_{1\left(2\right)}〉={\text{LG}}_{1\left(3\right)}^{5\left(-1\right)}$ , the SOP of two example states on the sphere are included as insets – as well as the intensity and phase of the polar spatial modes and their associated circular polarization handedness. (B) Schematic showing the separation of orthogonally polarized components from a vector state |Ψ⟩, by a Wollaston prism, followed by a complex amplitude hologram and their projection into the same diagonal plane using a linear polarizer.

Here, |*ψ*_*i*_⟩ represent arbitrary, un-normalized spatial modes, while |*R*, *L*⟩ represent the respective right- and left-circular polarization Jones matrices (these can be any two orthogonal Jones matrices – |*R*, *L*⟩ are chosen without loss of generality). The parameters *α* ∈ [0, *π*] and *β* ∈ [−*π*, *π*] represent the relative amplitudes and phases of the orthogonally polarized components. This state can be represented by a vector lying on the surface of the hybrid-order Poincaré sphere (HOPS) [32]. A HOPS is constructed with the scalar (i.e., *α* = 0, *π*) states on the poles and vector (i.e., *α* = *π*/2) states lying on the equator. The vector of a given field lying on the HOPS is specified by the same parameters *α* and *β* which are the respective polar and azimuthal coordinates. An example of a HOPS and two states lying on its surface are shown in Figure 1A (note that the tensor product operator ⊗ has been suppressed in the text for brevity). The ellipses overlaying the “non-scalar” fields represent the local state-of-polarization (SOP) across the transverse plane – where green ellipses are used to represent approximately linear polarization and red(blue) to represent right-(left-) circular handedness. From Eq. (1) and Figure 1A we can see that, in order to completely specify the state of a vector field, we need to know the spatial modes |*ψ*_1,2_⟩ as well as the parameters *α* and *β*. In order to determine |*ψ*_1,2_⟩, we can independently compute their inner products with the modes of a complete orthogonal basis (e.g., the Laguerre–Gaussian, LG, basis), according to:

(2) $$c_{p}^{l}=\left\langle {\text{LG}}_{p}^{l}|\psi_{1,2}\right\rangle ,$$

where *p* and *l* are the respective radial and azimuthal indices of the LG modes. If $c_{p}^{l}\neq 0$ for more than one ${\text{LG}}_{p}^{l}$ mode, then the relative phases $\phi_{p}^{l}$ between these modes and a reference mode ${\text{LG}}_{p_{ref}}^{l_{ref}}$ (with $c_{p_{ref}}^{l_{ref}}\neq 0$ ) can be acquired by executing their unbiased inner products

(3) $$d_{p}^{l}=〈{\text{LG}}_{p}^{l}+{\text{LG}}_{p_{ref}}^{l_{ref}}|\psi_{1,2}〉$$

(4) $$e_{p}^{l}=〈{\text{LG}}_{p}^{l}+i{\text{LG}}_{p_{ref}}^{l_{ref}}|\psi_{1,2}〉,$$

and then computing

(5) $$\phi_{p}^{l}=\arctan\left(\frac{2|e_{p}^{l}{|}^{2}-|c_{p_{ref}}^{l_{ref}}{|}^{2}-|c_{p}^{l}{|}^{2}}{2|d_{p}^{l}{|}^{2}-|c_{p_{ref}}^{l_{ref}}{|}^{2}-|c_{p}^{l}{|}^{2}}\right).$$

We can then reconstruct |*ψ*_1,2_⟩ according to

(6) $$|\psi_{1,2}〉=\underset{l,p}{\sum }c_{p}^{l}{\text{e}}^{\text{i}\phi_{p}^{l}}{\text{LG}}_{p}^{l}.$$

this procedure is known as modal decomposition [33].

With |*ψ*_1(2)_⟩ specified, we can now determine *α* and *β*. To begin, let us consider taking inner products of |Ψ⟩ with |*ψ*_1(2)_⟩|*D*⟩ individually (i.e., we project into the spatial mode and polarization degree of freedom simultaneously) – where we denote the intensities of these projections as *I*_1(2)_

(7) $$I_{1}=|〈\psi_{1}|〈D|\Psi 〉{|}^{2}=\frac{1}{2}\;\cos^{2}\left(\frac{\alpha }{2}\right)$$

(8) $$I_{2}=|〈\psi_{2}|〈D|\Psi 〉{|}^{2}=\frac{1}{2}\;\sin^{2}\left(\frac{\alpha }{2}\right).$$

Next, we can consider taking inner products with respect to superpositions of spatial modes |*ψ*_1(2)_⟩

(9) $$I_{3\left(4\right)}=|〈\psi_{1}\pm \psi_{2}|〈D|\Psi 〉{|}^{2}=\frac{1}{2}\pm \sin\left(\alpha \right)\cos\left(\beta \right)$$

(10) $$I_{5\left(6\right)}=|〈\psi_{1}\pm i\psi_{2}|〈D|\Psi 〉{|}^{2}=\frac{1}{2}\pm \sin\left(\alpha \right)\sin\left(\beta \right).$$

Using these six intensity measurements *I*_*i*_, we can construct the Cartesian coordinates Σ_*i*_ of the state vector |Ψ⟩

(11) $$\Sigma_{1}=I_{3}-I_{4}=2\;\sin\left(\alpha \right)\cos\left(\beta \right)$$

(12) $$\Sigma_{2}=I_{5}-I_{6}=2\;\sin\left(\alpha \right)\sin\left(\beta \right)$$

(13) $$\Sigma_{3}=I_{1}-I_{2}=\cos\left(\alpha \right).$$

Therefore, we can extract *α* and *β* as

(14) $$\alpha =\arctan\left(\frac{\Sigma_{3}}{\sqrt{\Sigma_{1}^{2}+\Sigma_{2}^{2}}}\right)$$

(15) $$\beta =\arctan\left(\frac{\Sigma_{1}}{\Sigma_{2}}\right).$$

A detailed derivation of the expression above is included in the Supplementary Information. The projection into the |*D*⟩ polarization allows for the exploitation of the third Arago–Fresnel law which states “Orthogonally polarized light derived from polarized light, when brought into the same plane, will interfere” [34]. From Eqs. (14) and (15) we can see how only six intensity measurements are required to extract global parameters *α* and *β*. Additionally, by simply replacing the spatial mode projections with identity projections, we recover spatially varying Stokes intensity measurements.

## 3 Experimental procedure

### 3.1 Generation

**Figure 2:**
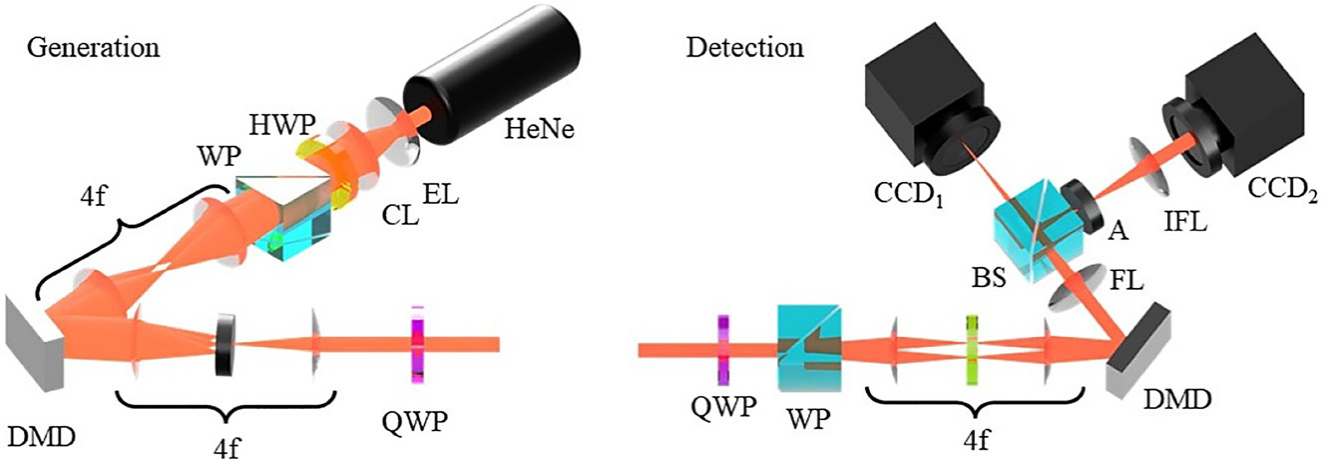
(Left) Diagram showing the arrangement used to generate arbitrary vector beams using a Wollaston prism to separate orthogonal circular polarizations and a DMD to recombine these – post modulation. (Right) Diagram showing the arrangement used to characterize arbitrary vector fields making use of a Wollaston prism to separate and a DMD to recombine orthogonal polarization components, with a linear polarizer to allow for interference of orthogonally polarized components. Abbreviations: HeNe – helium neon laser, EL – expansion lens, CL – collimation lens, HWP – half-wave plate, WP – Wollaston prism, BS – beam splitter, LP – linear polarizer, DMD – digital micro-mirror device, FL – Fourier lens, IFL – inverse Fourier lens, CCD – charge coupled device.

In order to test our detection scheme on a variety of vector beams we implemented a generation process which used the arrangement shown in (the left of) Figure 2 [17]. A Gaussian beam from a 632.8 nm HeNe laser was expanded and collimated using lenses EL and CL respectively – such that the central region approximated a constant intensity plane wave. A half-wave plate (HWP) was used to change the plane of polarization to diagonal. The expanded beam was passed through a WP (splitting the horizontally and vertically polarized components at an angle of ≈1° ). A 4*f* imaging system was used to image the plane at the WP on to the screen of a DMD (TI-DLP6500). The DMD was encoded with multiplexed binary holograms of the form [35]

(16) $$\begin{array}{ll} H_{A\left(B\right)} & =\frac{1}{2}+\frac{1}{2}\mathrm{sign}\left(\cos\left(2\pi G_{A\left(B\right)}\left(r\right)+\pi \Phi_{A\left(B\right)}\left(r\right)\right)\right.\\ & \;\left.-\cos\left(\pi A_{A\left(B\right)}\left(r\right)\right)\right), \end{array}$$

Here, $G_{A\left(B\right)}\left(r\right)=g_{A\left(B\right)}^{x}x+g_{A\left(B\right)}^{y}y$ are gratings with spatial carrier frequencies $g_{A\left(B\right)}^{x,y}$ . The 0th diffraction orders of the two orthogonally polarized beams leaving the DMD do so at the 1° separation angle; therefore, it is possible to pick $g_{A\left(B\right)}^{x,y}$ such that the 1st diffraction orders of each polarization component modulated by either *H*_*A*_ or *H*_*B*_ perfectly overlap [17]. $\Phi_{A\left(B\right)}\left(r\right)=\arg\left(T_{A\left(B\right)}\left(r\right)\right)/\pi$ and *A*_*A*(*B*)_(**r**) = arcsin (|*T*_*A*(*B*)_(**r**)|)/*π* are normalized phase and amplitude modulations of any desired complex transmission function *T*_*A*(*B*)_(**r**). A 4*f* imaging system was used to image the beam produced by the generation DMD into the detection setup (with an aperture at the focal plane to spatially filter the desired combined 1st diffraction order and a quater-wave plate [QWP] to convert horizontal and vertical linear polarization to left- and right-circular, respectively). Compensating aberration correction and holographic scaling (following beam shaping best practice [36]) was an applied to this beam prior to passing it through the detection system. A brief description of the holographic scaling and aberration correction are given in the Supplementary Information. This technique allowed for the generation of high quality arbitrary vector fields (e.g., by setting $T_{A\left(B\right)}={\text{LG}}_{1\left(3\right)}^{5\left(-1\right)}$ we can generate the vector field shown in Figure 1).

### 3.2 Detection

The core device – the detection system (shown on the right of Figure 2), was arranged with a QWP placed before a WP positioned at the plane of interest, which – together – separated the right- and left-circularly polarized components of the input field at an angle of 1°. A 4*f* imaging system was used to image this plane onto the screen of a DMD (TI-DLP4710). A diagonally orientated linear polarizer (LP) was placed within the 4*f* system in order to acquire projections of orthogonal polarizations in the same plane. The detection DMD was also addressed with multiplexed complex amplitude holograms of the form in Eq. (16). The Fourier intensity of the modulated combined beam at the DMD was imaged onto a CCD_1_ using a Fourier lens (FL), measuring on-axis intensities at this plane together with appropriate *T*_*A*(*B*)_ allowed for determination of inner products [33]. A 50:50 beam splitter (BS) was used to capture the image plane intensity using a second CCD_2_ after an aperture (*A*) and an inverse Fourier lens (IFL). In Table 1 we show the transmission functions *T*_*A*(*B*)_ used to acquire $c_{p}^{l}$ , $d_{p}^{l}$ , $e_{p}^{l}$ and *I*_*i*_ measurements in order to completely characterize the vector fields. The top segment of Table 1 has the DMD encoded with transmission functions to decompose the right-/left-circularly polarized fields into ${\text{LG}}_{p}^{l}$ modes, the middle segment contains the transmission functions to extract global parameters *α* and *β*, while the bottom segment has transmission functions to extract four Stokes intensity (*I*_*R*_, *I*_*L*_, *I*_*H*_ and *I*_*D*_) measurements used to extract the SOP and VQF [18, 28, 37]. The VQF is extracted according to

(17) $$VQF=Re\left(\sqrt{1-\frac{s_{1}^{2}+s_{2}^{2}+s_{3}^{2}}{s_{0}^{2}}}\right)$$

where *s*_*i*_ = *∫S*_*i*_(**r**)d**r** are the global Stokes parameters, with *S*_*i*_ being the spatially varying Stokes parameters determined as

(18) $$S_{0}=I_{R}+I_{L}$$

(19) $$S_{1}=2I_{H}-S_{0}$$

(20) $$S_{2}=2I_{D}-S_{0}$$

(21) $$S_{3}=I_{R}-I_{L}$$

**Table 1:** Table showing the transmission functions *T*_*A*(*B*)_ used to acquire measurements to specify: orthogonal circularly polarized components of a vector field (top), the relative amplitudes and phases of the orthogonal components (middle) and the Stokes intensity measurements (bottom).

T_A_	T_B_	CCD	Component	Measurement
${\left({LG}_{p}^{l}\right)}^{*}$	0	1	| ***ψ***_**1**_⟩	$c_{p}^{l}$
0	${\left({LG}_{p}^{l}\right)}^{*}$	1	***|ψ***_**2**_⟩	$c_{p}^{l}$
${\left({LG}_{p}^{l}+{LG}_{p_{\mathrm{ref}}}^{l_{\mathrm{ref}}}\right)}^{*}$	0	1	| ***ψ***_**1**_⟩	$d_{p}^{l}$
0	${\left({LG}_{p}^{l}+{LG}_{p_{\mathrm{ref}}}^{l_{\mathrm{ref}}}\right)}^{*}$	1	| ***ψ***_**2**_⟩	$d_{p}^{l}$
${\left({LG}_{p}^{l}+{iLG}_{p_{\mathrm{ref}}}^{l_{\mathrm{ref}}}\right)}^{*}$	0	1	| ***ψ***_**1**_⟩	$e_{p}^{l}$
0	${\left({LG}_{p}^{l}+{iLG}_{p_{\mathrm{ref}}}^{l_{\mathrm{ref}}}\right)}^{*}$	1	| ***ψ***_**2**_⟩	$e_{p}^{l}$
${\left(|\psi_{1}〉\right)}^{*}$	0	1	| ***ψ***_**1**_⟩	**I**_**1**_
0	${\left(|\psi_{2}〉\right)}^{*}$	1	| ***ψ***_**2**_⟩	**I**_**2**_
${\left(|\psi_{1}〉\right)}^{*}$	${\left(|\psi_{2}〉\right)}^{*}$	1	| ***ψ***_**1**_⟩ & | ***ψ***_**2**_⟩	**I**_**3**_
${\left(|\psi_{1}〉\right)}^{*}$	$e^{i\pi }{\left(|\psi_{2}〉\right)}^{*}$	1	| ***ψ***_**1**_⟩ & | ***ψ***_**2**_⟩	**I**_**4**_
${\left(|\psi_{1}〉\right)}^{*}$	$e^{i\pi /2}{\left(|\psi_{2}〉\right)}^{*}$	1	| ***ψ***_**1**_⟩ & | ***ψ***_**2**_⟩	**I**_**5**_
${\left(|\psi_{1}〉\right)}^{*}$	$e^{\mathrm{i3}\pi /2}{\left(|\psi_{2}〉\right)}^{*}$	1	| ***ψ***_**1**_⟩ & | ***ψ***_**2**_⟩	**I**_**6**_
**1**	**0**	2	| ***ψ***_**1**_⟩	**I**_**R**_
**0**	**1**	2	| ***ψ***_**2**_⟩	**I**_**L**_
**1**	**1**	2	| ***ψ***_**1**_⟩ & | ***ψ***_**2**_⟩	**I**_**H**_
**1**	**e** ^***iπ/2***^	2	| ***ψ***_**1**_⟩ & | ***ψ***_**2**_⟩	**I**_**D**_

## 4 Results

**Figure 3:**
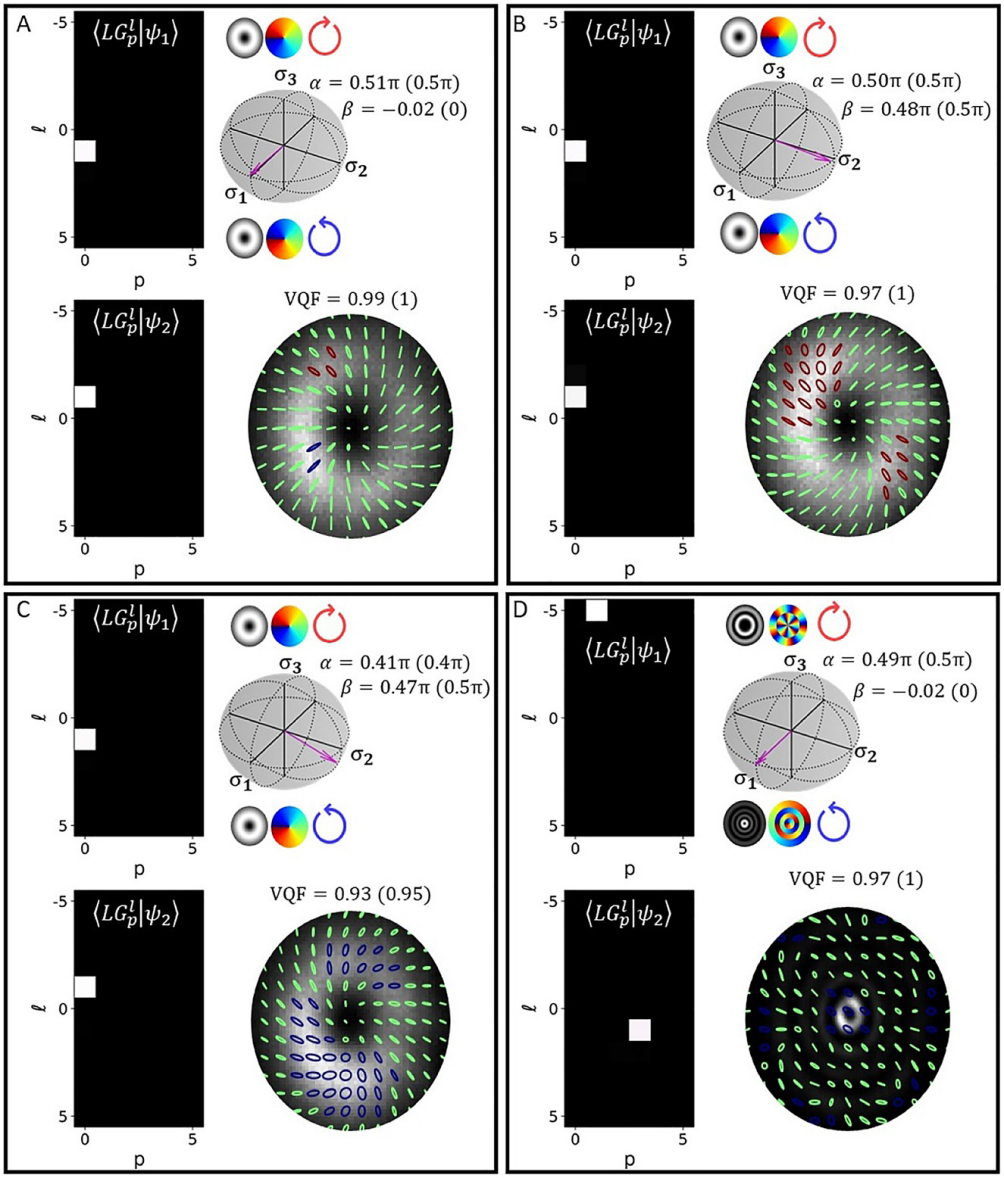
Crosstalk matrices (left), HOPS spheres with associated state vectors (top right) and SOP (bottom right) – measured for beams generated with (A) *α* = *β* = 0, $|\psi_{1\left(2\right)}〉={\text{LG}}_{0\left(0\right)}^{1\left(-1\right)}$ (B) *α* = 0, *β* = −*π*/2, $|\psi_{1\left(2\right)}〉={\text{LG}}_{0\left(0\right)}^{1\left(-1\right)}$ (C) *α* = −0.1, *β* = −*π*/2, $|\psi_{1\left(2\right)}〉={\text{LG}}_{0\left(0\right)}^{1\left(-1\right)}$ and (D) *α* = *β* = 0, $|\psi_{1\left(2\right)}〉={\text{LG}}_{1\left(3\right)}^{-5\left(1\right)}$ . The ideal values for *α*, *β* and VQF are included in brackets to the right of the measured values.

As an initial demonstration of arbitrary vector state measurements, we generated cylindrical vector vortex beams (i.e., $|\psi_{1\left(2\right)}〉={\text{LG}}_{0}^{\pm 1}$ ), and varied the relative amplitude and phases (i.e., *α* and *β*) of the generated beams to determine how accurately these parameters can be extracted from our detection system. Additionally, each measurement was preceded by a decomposition of |*ψ*_1(2)_⟩ into (a subset of) the ${\text{LG}}_{p}^{l}$ basis in order to ensure the measurements are occurring on the correct HOPS (here we approximated any ${\text{LG}}_{p}^{l}$ mode containing >90% of the total power as |*ψ*_1,2_⟩). In Figure 3 we can see the modal cross-talk matrices for |*ψ*_1(2)_⟩ and the state vectors represented on the appropriate HOPS sphere with *α* and *β* compared to the ideal values in brackets. Also included in Figure 3 are spatially varying SOP and VQF measurements. In Figure 3D we show the results obtained for $|\psi_{1\left(2\right)}〉={\text{LG}}_{1\left(3\right)}^{-5\left(1\right)}$ to demonstrate our technique’s viability for the accurate characterisation of radial orders. We can note some slight vertical stretching of the SOP results, this is due to the DMD diffracting light at 12° to its surface normal (computational re-scaling of the Stokes intensities could alleviate this artefact – see Supplementary Information). The results show good agreement with the ideal values.

**Figure 4:**
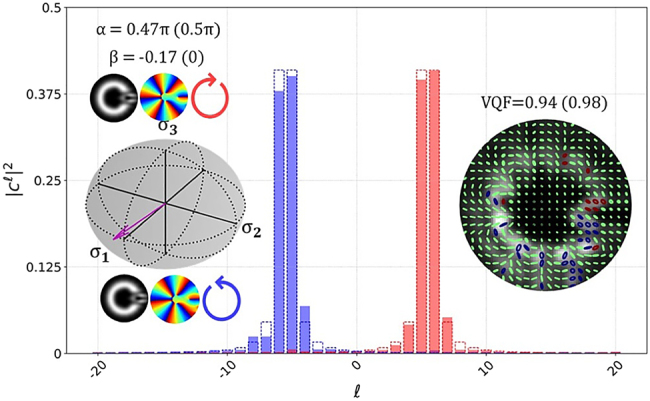
OAM spectra for individual components |*ψ*_1(2)_⟩ = e^±*i*5.5*φ*^ (red – |*ψ*_1_⟩, blue – |*ψ*_2_⟩; experimental results in solid bars and theoretical prediction in dashed lines). The vector on the HOPS sphere and SOP for the vector beam generated by the orthogonal fractional OAM components are included as insets.

In Figure 4 we present results, displaying our technique’s performance in characterizing vector beams made up of orthogonally polarized components with fractional OAM. The beams were generated by encoding *T*_*A*(*B*)_ = e^±*iφM*^ on the generation DMD, where *φ* is the azimuthal coordinate and *M* = *m* + *μ* is a fractional OAM index, with m∈Z and *μ* ∈ (0, 1). This generation hologram behaves like a spiral phase plate with a non-integer winding number. It has been shown that beams generated by such systems can be approximated as a finite superposition of beams with integer OAM, weighted by complex coefficients *f*_*l*_ calculated as [38], [39], [40]

(22) $$f_{l}=\left(\frac{i}{2\pi \left(M-l\right)}\right)\left(1-{\text{e}}^{\text{i}\left(2\pi \mu \right)}\right).$$

It should be noted that factors of the above expression relating the orientation of the phase discontinuity to the intra-modal phases have been omitted for brevity. We also adjusted our detection scheme to that of a phase only OAM decomposition, this avoids the need to sample the variety of radial orders which naturally manifest from phase plates (interested readers are directed to Refs [39, 41]). The OAM decomposition involves setting transmission functions equal to e^−*ilφ*^, since these are not eigenmodes of free space the physical effect is measuring the total power of ${\text{LG}}_{p}^{l}$ modes for all values of *p* (truncated by the DMD screen size) – therefore, the resulting OAM spectrum gives only the weightings of spiral phases (with charge *l*) in a given field. The OAM spectra of the |*ψ*_1_⟩ (red) and |*ψ*_2_⟩ (blue) components show good correlation with the theoretical predictions (dashed). The HOPS inset demonstrates the relative stability of the system in characterizing vector coordinates, while the SOP inset reveals the detail captured by the Stokes intensity measurements. It is notable that the VQF of this beam is lower than unity, this is due to the modal crosstalk evident in the OAM spectrum.

**Figure 5:**
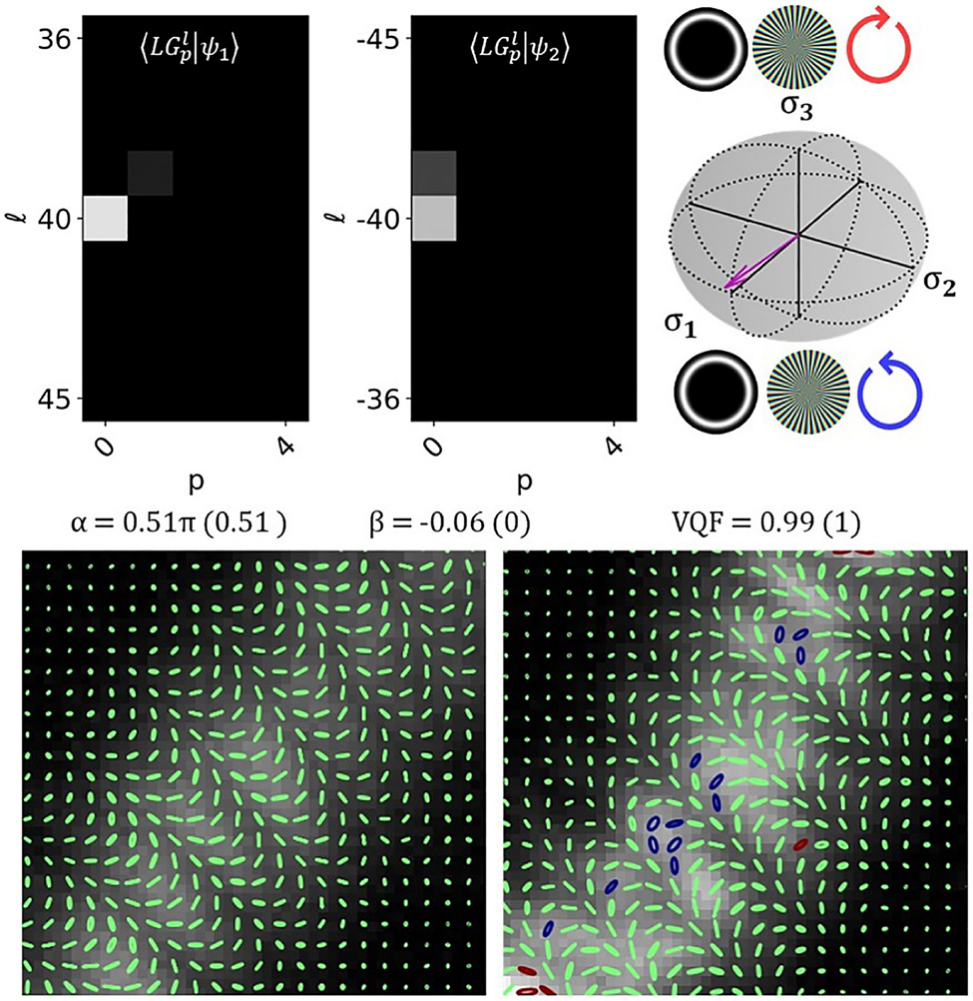
Crosstalk matrices and state vector lying on the HOPS sphere for $|\psi_{1\left(2\right)}〉={\text{LG}}_{0\left(0\right)}^{40\left(-40\right)}$ (top left). HOPS sphere for *l* ± 40 with the state vector (top right). Cropped regions displaying the SOP.

In Figure 5 we can see results obtained for beams with *l* = ±40. This represents the largest mode order our system is able to analyse (with a beam waist of *w*_0_ = 0.6 mm). This limitation of the system is determined by the size of the DMD and/or WP which impedes detection of beams with a larger spatial extent, this limit will generally precede the mode fidelity limit of the DMD which has been shown to exceed that of SLMs [42]. The crosstalk matrices presented, represent a portion of the entire *l* ∈ [−50, 50], *p* ∈ [0, 10] subspace which was sampled. The power in the desired ${\text{LG}}_{0}^{\pm 40}$ for |*ψ*_1(2)_⟩ was 0.883(0.739). The power dispersion arises due to larger mode orders being more sensitive to phase aberrations as well as on-axis centering at CCD_1_, choosing an FL with a longer focal length is expected to improve the performance. This result, however, still implies our device’s ability to reliably analyze cylindrically symmetric vector fields with *M* = |*l*| + 2p ≤ 40; as is revealed by the state vector displayed on the *l* = ±40 HOPS. Additionally the image plane Stokes intensities allowed for the recovery of the detailed inhomogenous SOP distribution embedded in this beam.

## 5 Conclusions

We have presented our arrangement, composed of a WP to separate orthogonal polarization components, a DMD to modulate and recombine them as well as a static linear polarizer to project the orthogonally polarized components into the same plane and interfere. We have demonstrated the effectiveness of our approach in completely specifying the state of a vector field, as well as in reconstructing the SOP of the field along with the associated VQF. We have shown how we can accurately characterise cylindrical vector vortex beams, vector beams with radial orders and vector beams with orthogonal components containing fractional OAM while also being able to extract digital Stokes intensity measurements. Additionally our system is able to characterize a significantly large subset of mode orders while maintaining a more compact arrangement while avoiding the need for reference fields, therefore addressing some of the problems faced by preceding vectorial characterization techniques. Furthermore the dynamic nature of the arrangement as well as the dual Fourier and image plane sampling leaves room for extensions to the functionality of our scheme. We believe this scheme will be of interest in industry, particularly for the characterization of liquid crystal and metasurface q-plates and j-plates, as well as other areas such as communications, microscopy and laser machining – where vectorial structured light is seeing increasing interest.

## Supplementary Material

Supplementary Material Details
