# Supplementary material for: Digital toolbox for vector field characterization
Source: Nanophotonics. 2021 Dec 7;11(4):753–61. doi: 10.1515/nanoph-2021-0484 (PMC11501183; doi:10.1515/nanoph-2021-0484)
Supplement: Supplementary file 1 — Supplementary Material Details [file j_nanoph-2021-0484_suppl.pdf]

Supplementary Information

Keshaan Singh and Angela Dudley*

Supplementary information: Digital toolbox for vector field characterization

https://doi.org/xx, Received xx; accepted xx

PACS: ...

Communicated by: xx

1 Derivation for relative global amplitude and phase extraction

Consider the vector field given by

$$|\Psi\rangle = \cos\left(\frac{\alpha}{2}\right) e^{i\frac{\beta}{2}} |\psi_1\rangle |R\rangle + \sin\left(\frac{\alpha}{2}\right) e^{-i\frac{\beta}{2}} |\psi_2\rangle |L\rangle, \tag{1}$$

where α and β are the respective global relative amplitude and phase parameters, $|\psi_{1(2)}\rangle$ represent arbitrary orthogonal spatial modes and $|R(L)\rangle$ represent the right-/left-circular polarization Jones vectors. Now let us consider the field resulting from simultaneous projections performed by our detection system

$$(c_1\langle D|\langle\psi_1| + c_2\langle D|\langle\psi_2|)|\Psi\rangle \tag{2}$$

$$= c_1 \cos\left(\frac{\alpha}{2}\right) e^{i\frac{\beta}{2}} (\langle\psi_1|\psi_1\rangle\langle D|D\rangle + i\langle\psi_1|\psi_1\rangle\langle D|A\rangle) \tag{3}$$

$$+ c_2 \cos\left(\frac{\alpha}{2}\right) e^{i\frac{\beta}{2}} (\langle\psi_2|\psi_1\rangle\langle D|D\rangle + i\langle\psi_2|\psi_1\rangle\langle D|A\rangle) \tag{4}$$

$$+ c_1 \sin\left(\frac{\alpha}{2}\right) e^{i\frac{\beta}{2}} (\langle\psi_1|\psi_2\rangle\langle D|D\rangle - i\langle\psi_1|\psi_2\rangle\langle D|A\rangle) \tag{5}$$

$$+ c_2 \sin\left(\frac{\alpha}{2}\right) e^{i\frac{\beta}{2}} (\langle\psi_2|\psi_2\rangle\langle D|D\rangle - i\langle\psi_2|\psi_2\rangle\langle D|A\rangle) \tag{6}$$

$$= c_1 \cos\left(\frac{\alpha}{2}\right) e^{i\frac{\beta}{2}} + c_2 \sin\left(\frac{\alpha}{2}\right) e^{-i\frac{\beta}{2}}, \tag{7}$$

Keshaan Singh, Angela Dudley, School of Physics, University of the Witwatersrand, Private Bag 3, Johannesburg 2050, South Africa

Label	c_1	c_2	Result
I_1	1	0	$\cos^2\left(\frac{\alpha}{2}\right)$
I_2	0	1	$\sin^2\left(\frac{\alpha}{2}\right)$
I_3	1	1	$1 + 2\sin(\alpha)\cos(\beta)$
I_4	1	-1	$1 - 2\sin(\alpha)\cos(\beta)$
I_5	1	i	$1 + 2\sin(\alpha)\sin(\beta)$
I_1	1	$-i$	$1 - 2\sin(\alpha)\sin(\beta)$

Tab. 1: Table showing the coefficients $c_{1(2)}$ used to take the six intensity measurements required to extract global relative amplitude and phase information.

where $|D(A)\rangle$ represent the diagonal/anti-diagonal linear polarization Jones vectors. To simplify Eqn 7 we exploit the orthogonality of both the polarization and spatial mode bases (i.e. $\langle\psi_i|\psi_j\rangle = \delta_{ij}$, $\langle D|D\rangle = 1$ and $\langle D|A\rangle = 0$). $c_{1(2)}$ are complex constants which can be introduced by the DMD in conjunction with the spatial mode correlation filter. We can then determine a set of six intensity measurements I_i characterized by the coefficients $c_{1(2)}$ given in Tab. 1

The expressions in Tab. 1 have been calculated by taking the squared absolute value of Eqn 7 and reduced using appropriate trigonometric identities. From these six intensity measurements we can construct the Cartesean components of the hybrid order sphere on which the vector field $|\Psi\rangle$ lies

$$\Sigma_1 = I_3 - I_4 = 4\sin(\alpha)\cos(\beta) \tag{8}$$

$$\Sigma_2 = I_5 - I_6 = 4\sin(\alpha)\sin(\beta) \tag{9}$$

$$\Sigma_3 = I_1 - I_2 = \cos(\alpha). \tag{10}$$

It is notable that Σ_i represent the expectation values of the Pauli matrices, σ_i, with respect to a global Jones vector $\Gamma = (\cos\left(\frac{\alpha}{2}\right) e^{i\frac{\beta}{2}}\ \sin\left(\frac{\alpha}{2}\right) e^{-i\frac{\beta}{2}})^T$ (i.e. $\Sigma_i = \langle\Gamma|\sigma_i|\Gamma\rangle$). This is an expected result, as we are dealing with a bipartite (two degrees-of-freedom) two dimensional system. Naturally we can then extract the spherical coordinates α and β according to

$$\alpha = \arctan\left(\frac{\sigma_3}{\sqrt{\sigma_1^2 + \sigma_2^2}}\right) \tag{11}$$

$$\beta = \arctan\left(\frac{\sigma_1}{\sigma_2}\right). \tag{12}$$

This result shows how our scheme is capable of retrieving global relative amplitude and phase information from vector fields using simultaneous spatial

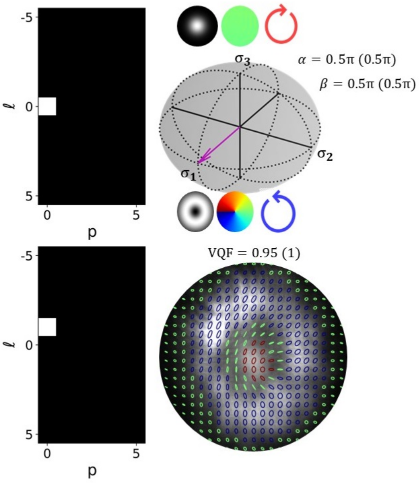

Fig. 1: Results obtained from modal decomposition measurements (left), global parameter measurements (top right) and Stokes intensity measurements (bottom right) performed on a full-Poincaré beams.

mode and polarization projective measurements, which is used to specify the state vector $|\Psi\rangle$ on the hybrid order Poincaré sphere.

2 Full-Poincaré beams

Full-Poincaré beams have transverse polarization profiles which contain approximately all possible states of polarization (i.e. all points on the Poincaré sphere stereographically projected onto the transverse plane). These exotic beams can be easily constructed using the Laguerre-Gaussian basis modes according to

$$|\Psi\rangle = LG_0^0|R\rangle + LG_0^1|L\rangle, \tag{13}$$

where we are considering the simple case where $\alpha = \frac{\pi}{2}$ and $\beta = 0$. Naturally these beams fall into the detectable range of mode orders our scheme is capable of resolving. The results obtained from modal decomposition, global parameter extraction and Stokes measurements - performed on a full-Poincaré beam of the form in Eqn 13 - are given in Fig. 1

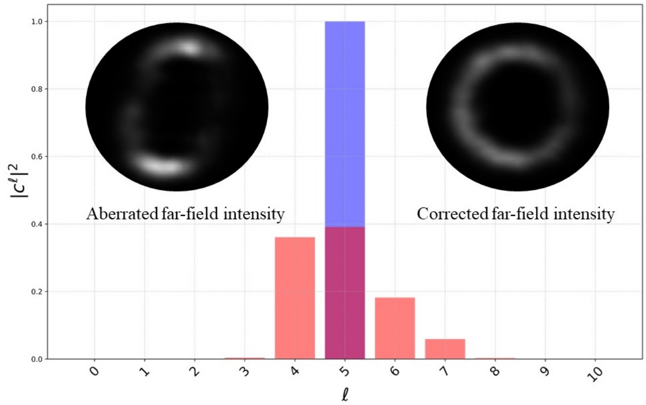

Fig. 2: OAM spectrum measured for a scalar LG_0^5 beam with (blue) and without (red) holographic aberration correction. Far-field intensity measurements of the aberrated and corrected beams are included as insets.

3 Aberration correction employed by a DMD

Optical systems, in general, will be subject to aberrations due to manufacturing error and misalignment as well as stress induced aberrations in soldered opto-electronic components such as DMD screens. These aberrations are conventionally and easily described in terms of linear combinations of the Zernike polynomials Z_n^m, which are normalized on a disk of arbitrary radius (as the coordinates can be scaled to treat the radius as unity). For aberrations from the sources discussed above, the magnitude of the coefficients scale inversely with the polynomial order - therefore the first six non-trivial (i.e. excluding the constant phase Z_0^0) polynomials can be used to effectively approximate these aberrations. This means that simply introducing the conjugate of the aberration into the holographic transmission function of the DMD can result in approximately unaberrated beams. In Fig. 2 we can see the effect of aberrations on the far field intensity of a scalar LG_0^5 mode as well as the measured OAM spectrum detected from this mode - we also display how the holographic correction improves the mode quality.

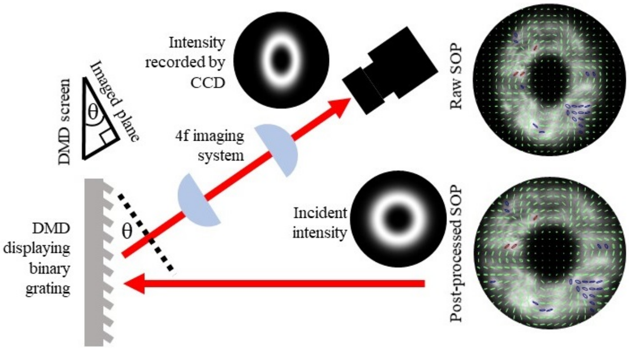

Fig. 3: Diagram showing the origin of horizontal scaling due to the DMD diffraction angle. The SOP measured for $|\Psi\rangle = LG_0^5|R\rangle + LG_0^{-5}|L\rangle$ with (bottom) and without (top) compensating post-processing are included as insets.

4 Accounting for large diffraction angles when working with DMDs

Generally, a $4f$ imaging system with a spatially filtering iris placed at the focus is used to extract a complex field encoded into the first diffraction order by a DMD. The diffraction grating is generated by actuating the micro-mirrors by angles $\pm\theta$, where the positive angles can be associated with transmitting portions of a grating and negative angles with completely absorbing portions (referred to as the on and off states). Consequently, the optical axis of the desired light is reflected from the DMD surface at the angle θ. This angular diffraction causes the imaging of a plane offset from the DMD screen by θ, a diagram illustrating the origin in impact of this artefact is given in Fig. 3

If light is being shaped from a uniform plane wave, then compensation for this effect can be made within the hologram. However, as shown in Fig. 3, if shaped light is being modulated (e.g. by a pure phase grating) then post-processing of the CCD image needs to be employed to account for the scaling. The holographic compensation involves changing the real space dimensions of each pixel according to $dy \times dx$ m $\rightarrow dy \times \cos(\theta)dx$ m when creating the transmission functions. The post-processing compensation can be achieved by reshaping (using a suitable interpolation technique) the captured $N \times M$ pixel image to a $N \times \frac{M}{\cos(\theta)}$ pixel image.

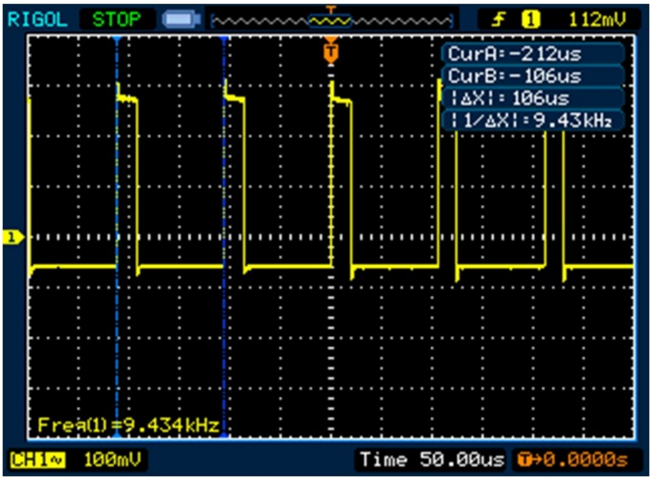

Fig. 4: Image showing the waveform produced by a DMD trigger output, measured by an oscilloscope.

5 Time resolution

In Fig. 4 we can see the waveform of the trigger signal produced by a DMD (Texas Instruments-DLP6500) recorded by an oscilloscope (RIGOL-DS1052E), each pulse corresponds to a single binary image displayed by the DMD screen. We can see that binary images are displayed at 9.43 kHz. This refresh rate exceeds the acquisition rates of most CCD cameras ($\approx$ 149 Hz), but can be matched by photodiode arrangements. This means that the Stokes intensity acquisition rate is limited by the CCD rate according to $f_{Stokes} = \frac{149}{4} = 37.25$ kHz; while the spatial mode projective measurements (e.g. a modal decomposition of two polarization states into $N = 50$ modes, as well as the intra modal phase measurements) are limited by the DMD rate according to $f_{Projection} = \frac{9.43 \times 10^3}{6N-4} = 31.85$ Hz.
